# Competence and confidence of early-career psychiatrists in the assessment and management of negative symptoms

**DOI:** 10.1192/j.eurpsy.2026.10354

**Published:** 2026-07-31

**Authors:** A. J. Krupa

**Affiliations:** Department of Biological and Community Psychiatry, Jagiellonian University Medical College, Krakow, Poland

## Abstract

**Abstract:**

Negative symptoms of schizophrenia represent a major determinant of long-term functional impairment and quality of life, yet they remain insufficiently recognized and inadequately managed in routine clinical practice. Although appropriate brief assessment instruments and international guidance papers have been developed over the past two decades, their effective integration into psychiatric training and everyday care has not yet been verified. This study aimed to evaluate the level of knowledge and self-perceived competence in evaluating and managing of negative symptoms among early career psychiatrists (ECPs) in Europe.

The CARE project was a cross-sectional online survey conducted between January and June 2025 and distributed through European psychiatric networks. A total of 828 ECPs from 19 European countries were included in the analysis. The survey assessed demographic and training-related characteristics, knowledge and familiarity with guideline-based recommendations, and self-reported competence in evaluation and treatment of negative symptoms.

Despite high self-reported exposure to theoretical courses about negative symptoms and clinical placements in schizophrenia care wards/clinics (67.9% and 70.3%) and 65.7% of ECPs reporting feeling well-trained to administer negative symptom assessment tools, the objective knowledge of negative symptoms was limited. Only 11% of participants correctly identified all core negative symptom domains while 15.9% provided correct answers to European Psychiatric Association guidance-based questions. In contrast, a considerably larger percentage of 46.7% and 25.9% respondents reported feeling competent in the evaluation and management of negative symptoms, indicating a striking discrepancy between perceived competence and actual knowledge about negative symptoms. Higher knowledge levels were associated with specialist status, engagement in clinical research, and participation in extracurricular training in negative symptoms, but not with the standard components of specialist curricula (theoretical courses and placements in wards/clinics specialized in schizophrenia care). Almost 90% of ECPs agreed that there should be more emphasis on negative symptoms in the specialist training curricula.

These findings highlight substantial educational gaps in the field of negative symptoms among European ECPs and suggest that current training programs need to be updated and refined to foster guideline-concordant expertise. Integrating evidence-based recommendations into mandatory curricula and providing ECPs with skills-oriented training may be the essential steps toward raising the recognition and management of negative symptoms and eventually improving the functioning and quality of life of people living with schizophrenia.

**Disclosure of Interest:**

None Declared

